# Community Socioeconomic Status and Dispatcher-Assisted Cardiopulmonary Resuscitation for Patients with Out-of-Hospital Cardiac Arrest

**DOI:** 10.3390/ijerph18031207

**Published:** 2021-01-29

**Authors:** Ching-Fang Tzeng, Chien-Hsin Lu, Chih-Hao Lin

**Affiliations:** 1 Harvard T. H. Chan School of Public Health, Boston, MA 02115, USA; tiffanytzeng1026@gmail.com; 2 Department of Emergency Medicine, Baylor Scott & White All Saints Medical Center, Fort Worth, TX 76104, USA; 3 Department of Emergency Medicine, National Cheng Kung University Hospital, College of Medicine, National Cheng Kung University, Tainan 70403, Taiwan; jazojazojazo@gmail.com

**Keywords:** dispatcher-assisted cardiopulmonary resuscitation, socioeconomic status, out-of-hospital cardiac arrest

## Abstract

Few studies have investigated the association between dispatcher-assisted cardiopulmonary resuscitation (DA-CPR) performance and the outcomes of out-of-hospital cardiac arrest (OHCA) among communities with different socioeconomic statuses (SES). A retrospective cohort study was conducted using an Utstein-style population OHCA database in Tainan, Taiwan, between January 2014 and December 2015. SES was defined based on real estate prices. The outcome measures included the achievement of return of spontaneous circulation (ROSC) and the performance of DA-CPR. Statistical significance was set at a two-tailed *p*-value of less than 0.05. A total of 2928 OHCA cases were enrolled in the high SES (*n* = 1656, 56.6%), middle SES (*n* = 1025, 35.0%), and low SES (*n* = 247, 8.4%) groups. The high SES group had a significantly higher prehospital ROSC rate, ever ROSC rate, and sustained ROSC rate and good neurologic outcomes at discharge (all *p* < 0.005). The low SES group, compared to the high and middle SES groups, had a significantly longer dispatcher recognition time (*p* = 0.004) and lower early (≤60 s) recognition rate (*p* = 0.029). The high SES group, but none of the DA-CPR measures, had significant associations with sustained ROSC in the multivariate regression model. The low SES group was associated with a longer time to dispatcher recognition of cardiac arrest and worse outcomes of OHCA. Strategies to promote public awareness of cardiac arrest could be tailored to neighborhood SES.

## 1. Introduction

Despite great efforts over the decades, out-of-hospital cardiac arrest (OHCA) remains a crucial issue in public health worldwide [1]. The overall survival rate of patients with OHCA is approximately 10–16% in the United States [2], and it is even lower among Asian countries, ranging from 0.5% to 8.5% [3,4]. Prehospital care could be associated with the survival outcomes of patients with OHCA, including emergency medical service (EMS) response time [5], initial cardiac rhythm [6], early defibrillation [7], bystander cardiopulmonary resuscitation (CPR) [8], and even socioeconomic factors [9].

Although both the incidence and mortality rate of OHCA have decreased in Taiwan in recent years, patients in remote areas still tend to have higher mortality [10]. The higher mortality of OHCA in remote areas could be associated with the inequality of regional EMS or medical resources [11]. Recent studies have found that the socioeconomic factors might be associated with bystander CPR rate and the outcomes of OHCA [12]. Communities with higher socioeconomic status (SES) could have higher bystander CPR rates and greater prognosis, which could be related to a larger percentage of people with knowledge of CPR and thus a greater likelihood of providing CPR efforts following resuscitation training [13,14].

Dispatchers are the first link to the EMS system [15]. DA-CPR through emergency calls can increase bystander CPR rates [16] and, subsequently, improve the survival rates and neurologic outcomes of OHCA [17,18]. Early recognition of cardiac arrest by dispatchers through emergency calls is essential for delivering effective CPR instructions to bystanders. The association of SES and performance of dispatcher-assisted cardiopulmonary resuscitation (DA-CPR) was rarely examined [19,20]. This study aimed to evaluate the DA-CPR performance of patients with OHCA occurring in different SES areas.

## 2. Methods

### 2.1. Study Design and Setting

We conducted a retrospective cohort study using an Utstein-style population database in Tainan City, Taiwan. Tainan City comprises an area of 2192 km^2^ with 37 administrative districts and a population of 1.9 million [21]. The EMS system of Tainan is fire-bureau-based. There are 52 EMS stations and 615 emergency medical technicians (EMTs) in Tainan city. The distribution of EMS stations and EMTs is mainly based on regulations and local population. The EMS dispatch center is centralized and single. The Taiwanese guidelines for prehospital CPR were modified from the American Heart Association, European Resuscitation Council, and International Liaison Committee on Resuscitation 2010 guidelines. Patients with cardiac arrest received resuscitation for at least two full cycles (approximately 5 min) on scene before being transported to a hospital. The resuscitation efforts continued during transport unless ROSC was achieved. At the same time, we did not have consistent rules for the termination of resuscitation in prehospital settings. As such, all patients with cardiac arrest who were assessed by EMTs were sent to hospitals unless obvious signs of irreversible death were present.

### 2.2. Selection of Participants

The enrollment criteria for patients with medical OHCA were those transported by the local EMS system between 1 January 2014 and 31 December 2015. Those with known pregnancies, those younger than eighteen years old, those with severe hypothermia or obvious signs of irreversible death, those with traumatic cardiac arrests, and those with valid do-not-attempt-resuscitate (DNAR) orders were excluded.

### 2.3. DA-CPR Program

Tainan City has adopted a comprehensive DA-CPR package since 1 June 2013 [21,22]. Dispatchers were trained to conduct a streamlined, two-step question approach to identify potential cardiac arrests. If the caller responded that the victim was unconscious and was not breathing normally, then the dispatcher initiated instructions for chest compression-only CPR. Voice recordings of emergency calls for patients with OHCA confirmed by EMTs on the scene were retrospectively retrieved and reviewed [21,22]. The details of the DA-CPR program in the Tainan City were discribed in our previous work [21].

### 2.4. Socioeconomic Status

SES is regarded as a mixture of people based on educational level, salary, occupation, or wealth; however, accurate evaluation of SES is challenging [19,20,23]. We assumed that the land value may reflect a more long-term evaluation of local SES. We, therefore, used the average land value in each district as a major measurement to evaluate the SES of the neighborhood, similar to other research studies [24,25,26,27].

The 37 districts in Tainan City were stratified into three groups by allocating those districts with the top 25% of average land value into the high SES group, those in the middle 50% of average land value into the middle SES group, and the lowest 25% of average land value into the low SES group. The data on the average land value, area, household incomes, and population of each district in Tainan were acquired from the 2016 annual report of Department of Land Administration and National Consensus database in Taiwan [28].

### 2.5. Data Collection and Processing

The data were obtained from a citywide OHCA registry database, including the dispatch registries, the EMS run registries, the EMS cardiac arrest registries, and an OHCA registry for hospital care and outcomes.

### 2.6. Outcome Measures

The primary outcome evaluated was defined as the achievement of a sustained (≥2 h) ROSC [29]. The secondary outcomes included prehospital ROSC, ever ROSC, survival at discharge, and good neurological status at discharge, defined by cerebral performance category (CPC) scale I or II.

The measurements of DA-CPR performance included dispatcher recognition of cardiac arrest, early dispatcher recognition of cardiac arrest (≤60 s), dispatcher initiation of bystander CPR, any dispatcher delivery of CPR instructions, and good quality of dispatcher delivery of CPR instructions until the arrival of EMTs.

Dispatcher recognition of cardiac arrest was defined as the presence of specific “cardiac arrest” voice-recording or the initiation of CPR instructions. The time of dispatcher recognition of cardiac arrest was defined as the time interval from taking the emergency call to the first signal representing dispatcher recognition of cardiac arrest. Early recognition of cardiac arrest was defined as the time of dispatcher recognition of cardiac arrest being no more than one minute. In this study, good quality of DA-CPR was defined as uninterrupted provision of CPR instructions to bystanders until the EMT’s arrival at scene. While the use of DA-CPR was emphasized during prehospital care for OHCA patients, the quality of DA-CPR was primarily dependent on communication between dispatchers and bystanders and the education levels of bystanders.

### 2.7. Primary Data Analysis

We used SPSS statistical software SPSS (version 17; SPSS Inc., Chicago, IL, USA) for the statistical analysis. Categorical variables are shown as numbers and percentages. Quantitative data are shown as mean values and standard deviations (SDs). The data distribution was examined with the Kolmogorov–Smirnov normality test. We used the Chi-squared test or Fisher’s exact test for categorical variables. The Mann–Whitney U test or the Kruskal–Wallis test was applied for continuous variables.

To identify independent associated factors for achievement of sustained ROSC, we first applied univariate logistic regression to obtain the associated odds ratios (ORs) and 95% confidence intervals (CIs). Subsequently, we constructed full models for multivariate logistic regression by including all of the major potential factors that were available in this study [29]. A two-tailed *p*-value less than 0.05 was considered statistically significant.

## 3. Results

### 3.1. Socioeconomic Status

The 37 districts in Tainan were stratified into the high SES (*n* = 10), middle SES (*n* = 17), and low SES (*n* = 10) groups based on the average price of real estate. During the study period, the average prices of real property in the high, middle, and low SES groups were 2740.9, 285.9, and 55.9 USD/m^2^, respectively. Among these three SES groups, the average price of real property, population, annual household incomes, and population density had significantly positive correlations with socioeconomic status, as shown in Table A1.

### 3.2. Patient Characteristics

**Figure 1:**
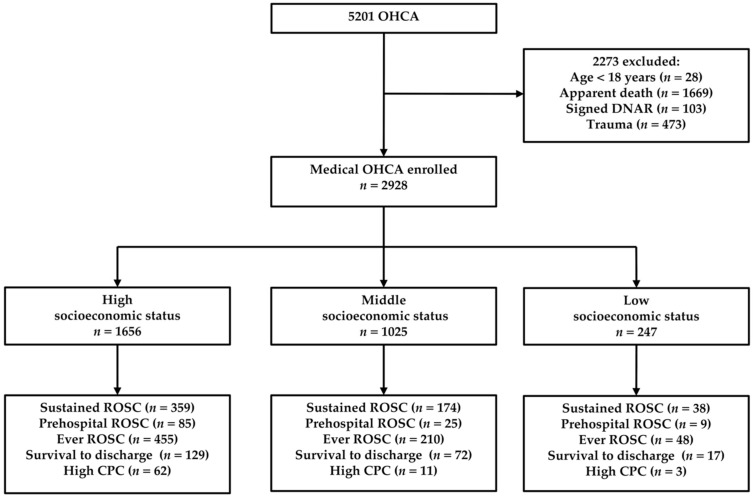
Overview of patients with OHCA during the study period. Abbreviations: CPC: cerebral performance category; DA-CPR: dispatcher-assisted cardiopulmonary resuscitation; DNAR: do-not-attempt-resuscitate; OHCA: out-of-hospital cardiac arrest.

A total of 5201 OHCAs assessed by EMTs in Tainan were evaluated for inclusion. The exclusion criteria were patients younger than 18 years old (*n* = 28), apparent death (*n* = 1669), patients who signed DNAR forms (*n* = 103), and traumatic OHCAs (*n* = 473). A total of 2928 OHCA cases were enrolled in the final analyses, of whom 1656 (56.6%) belonged to the high SES group, 1025 (35.0%) belonged to the middle SES group, and 247 (8.4%) belonged to the low SES group. Figure 1 provides an overview of the OHCA patients evaluated during the study period.

The characteristics of patients with OHCA in the three SES areas are shown in Table 1. OHCAs occurring in the high SES group were less likely to be witnessed (*p* < 0.001). The EMS response time and transportation time were significantly longer in the low SES group (*p* < 0.001). Among the three groups, the middle SES group had the highest bystander CPR rate (*p* = 0.004). The high SES group had significantly higher prehospital ROSC rates, ever ROSC rates, sustained ROSC rates, and good neurological status at discharge (all *p* < 0.005) but not a superior rate of survival to discharge (*p* = 0.718). Regarding the achievement of a sustained ROSC, there were significant differences between the high and middle SES groups (*p* = 0.004) and between the high and low SES groups (*p* = 0.023), but not between the middle and low SES group (*p* = 0.547).

### 3.3. DA-CPR

There were no significant differences among the three SES groups regarding the rate of dispatcher recognition of cardiac arrest, the rate of dispatcher initiation of CPR, or the rate of high quality of DA-CPR (all *p* > 0.05). The time to recognition of cardiac arrest by dispatchers was significantly longer in the low SES group (64.1 ± 76.4 s), compared to the high SES (43.9 ± 35.3 s) and middle SES (41.7 ± 37.1 s) groups (*p* = 0.004), as shown in Table 2. The rate of early dispatcher recognition of cardiac arrest (≤60 s) was significantly lower in the low SES group (21.9%), compared to the high SES (30.1%) and the middle SES (31.1%) groups (*p* = 0.029).

**Figure 2:**
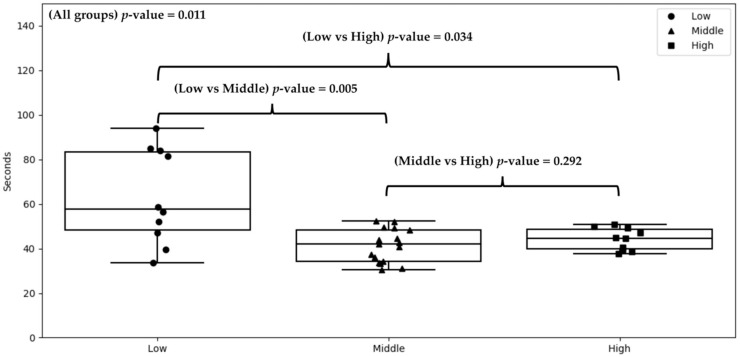
Average time of dispatcher recognition of cardiac arrest in the 37 districts among the three SES groups. The bars and boxes represented the range, median, and interquartile ranges of the average times among the different SES groups. Abbreviations: SES: socioeconomic status.

The average times of dispatcher recognition of cardiac arrest in the 37 districts among the three SES groups are depicted in Figure 2.

The analysis of obstacles that impeded DA-CPR is provided in Table A2. Language barriers had no significant difference among SES groups.

### 3.4. ROSC

Compared to OHCA patients without sustained ROSC, patients with sustained ROSC tended to have significantly less time for EMS response at the scene and transportation (all *p* < 0.05), as shown in Table 3.

Based on the above findings, we further examined the correlation between these factors and achievement of a sustained ROSC, as shown in Table 4. In the backward multivariate regression model, the high SES group (OR 1.54, 95% CI 1.06–2.23, *p* = 0.024) was significantly associated with sustained ROSC. However, none of the DA-CPR measures showed significant associations with sustained ROSC (all *p* > 0.05).

## 4. Discussion

In this study, we found that areas with low SES were significantly associated with longer times of dispatcher recognition of cardiac arrest, lower rates of early dispatcher recognition of cardiac arrest, and lower survival outcomes of OHCA.

Identifying the disparities in the performance of DA-CPR in a given community is essential for EMS system design. To the best of our knowledge, this study is the first to discuss the role of dispatcher recognition of cardiac arrest among different SES groups. Previous studies of SES and OHCA outcomes have been limited to events that occur in private residences, as these are more likely to reflect events that include residents of the community of interest. However, we considered the EMS calls as a collective behavior of a community and, therefore, we evaluated EMS calls for all OHCAs, either in private or public areas, in this study.

Many factors could hinder the dispatcher recognition of cardiac arrest. Language barriers can cause significant delays in initiating CPR [30]. The average time until telecommunicators recognized the CPR need was 87.4 s for the no language barrier group, compared to 160.6 s for the language barrier group [30]. Time until CPR instructions started was significantly different between the two groups, as was the time to first compression [30]. Lack of language proficiency could cause witnesses to prefer calling family members or friends, rather than calling emergency services numbers [31].

The use of emergency medical services varies among different language-speaking patients [32]. EMS providers should be prepared when responding to emergency calls involving patients with limited language proficiency [33]. A recent study found that the use of language might have an effect on CPR skills and willingness to intervene in OHCA [34]. Although Mandarin is the official language in Taiwan, more than 80% of Taiwanese people can speak both Mandarin and Hokkien, a Taiwanese traditional language, fluently [35]. All of the dispatchers in Tainan are fluent in either Mandarin or Hokkien. The dispatchers utilized a script-based protocol in both languages. Thus, we suspected that language is not the major barrier to having DA-CPR performance disparities among the three different SES groups in Tainan. In our analysis, only three emergency calls were related to language barriers, which were all involved with foreign caregivers (Table A2). No sound quality problems with the OHCA emergency calls were reported during the period.

A longer duration for understanding communication could be detrimental to the outcome of OHCA. We surmised that, aside from language barriers, sociocultural differences could influence the communication proficiency between the callers and the dispatchers and thus the time until recognition of cardiac arrest. The sociocultural difference and public awareness disparities might be reflected in the SES. The results of our study are consonant with recent findings that communities with higher public CPR awareness had higher rates of OHCA recognition during calls, DA-CPR, and bystander CPR [36].

OHCA patients in the higher SES group had higher rates of bystander CPR and even more prominent DA-CPR rates [20]. Bystander CPR with dispatcher assistance played an important role in OHCA that occurred in rural areas [19]. DA-CPR could enhance layperson performance of CPR before EMTs arrive, and untrained laypersons are instructed to properly compress the chest and follow guidance [15,37,38]. Although none of the DA-CPR measures exhibited significant associations with the survival outcomes of patients with OHCA in the multivariate model, the results of our study provided insights into DA-CPR programs for the future. We suggest that the strategies for better public knowledge of cardiac arrest be tailored to the neighborhood SES. The format and content of educational material deserve further investigation to effectively enhance public awareness. Although general CPR training has been greatly emphasized in resuscitation worldwide, establishment of standardized dispatcher assistance with several languages could improve survival outcomes efficiently. Additionally, recruitment of trained citizen volunteering for targeted communities with lower bystander CPR rates might improve the survival rates of OHCA patients [39,40].

## 5. Limitations

Our study has several limitations. First, this study was an analysis of OHCA registry with socioeconomic factors. Strong causal inferences between socioeconomic status and the quality of DA-CPR were beyond the purview of this study design. In Taiwan, it is convenient for people to travel among different cities, and visitors could have been included in our study. Further intensive epidemiologic research is needed to clarify the plausibility of our findings. Second, our research was conducted in a single city; thus, long-term and large-scale research is essential for validation of our findings. The distribution of EMS resources in the study city is mainly based on regulations and local population. Thus, the density of EMS stations/population and EMTs/population are similar in each district, but the density of EMS stations/area or EMTs/area could be varied largely. We did not evaluate the compositions of EMTs and medical resources in different SES areas [41]. Third, because SES is multifactorial, using real estate prices solely to evaluate SES could be imperfect. SES is composed of education, employment, income, and wealth [12]. Arrest location also plays a role in outcomes of DA-CPR. However, it is difficult to measure all of the facets of SES due to paucity of data. The use of real-estate value, as adopted from the previous literature, was an acceptable analysis [13,27]. Finally, the status of subjects staying in nursing homes, which may have more trained staff but be located in areas of low SES, is unknown. There was an assumption that the performance of the management of the accepting hospital to these patients is uniform. Additional research is needed to evaluate the role of communication in socioeconomic variables, such as family income and education [42].

## 6. Conclusions

The low SES group was associated with a longer time to dispatcher recognition of cardiac arrest and worse outcomes of OHCA. Strategies to promote public awareness of cardiac arrest could be tailored to neighborhood SES.

## Figures and Tables

**Table 1:** Characteristics in patients with out-of-hospital cardiac arrest by community socioeconomic status.

OHCA	High SES (*n* = 1656)	Middle SES (*n* = 1025)	Low SES (*n* = 247)	*p* Value
Age (years) (mean ± SD)	68.5 ± 16.7	70.6 ± 16.0	69.5 ± 14.5	0.010 *
Age (>65 years) (*n*, %)	984 (59.4%)	664 (64.8%)	156 (63.2%)	0.017 *
Male (*n*, %)	1056 (63.8%)	650 (63.4%)	161 (65.2%)	0.874
Public areas (*n*, %)	152 (9.2%)	88 (8.6%)	17 (6.9%)	0.475
Witnessed (*n*, %)	781 (47.2%)	567 (55.3%)	129 (52.2%)	<0.001 *
EMS Response time (min) (mean ± SD)	6.5 ± 2.6	8.3 ± 4.0	9.8 ± 5.8	<0.001 *
EMS Scene time (min) (mean ± SD)	10.6 ± 5.2	10.3 ± 9.0	10.6 ± 7.4	<0.001 *
EMS Transportation time (min) (mean ± SD)	6.0 ± 4.9	9.0 ± 5.4	14.4 ± 7.5	<0.001 *
High quality of DA-CPR (*n*, %)	306 (18.5%)	166 (16.2%)	38 (15.4%)	0.215
Shockable rhythm (*n*, %)	251 (15.2%)	141 (13.8%)	25 (10.1%)	0.092
Bystander CPR (*n*, %)	450 (27.2%)	316 (30.8%)	51 (20.6%)	0.004 *
Prehospital ROSC (*n*, %)	85 (5.1%)	25 (2.4%)	9 (3.6%)	0.003 *
Ever ROSC (*n*, %)	455 (27.5%)	210 (20.5%)	48 (19.4%)	<0.001 *
Sustained ROSC (*n*, %)	359 (21.7%)	174 (17.0%)	38 (15.4%)	0.003 *
Survival to discharge (*n*, %)	129 (7.8%)	72 (7.0%)	17 (6.9%)	0.718
Good neurologic outcome at discharge (*n*, %)	62 (3.7%)	11 (1.1%)	3 (1.2%)	<0.001 *

Abbreviations: SES: socioeconomic status; CPR: cardiopulmonary resuscitation; DA-CPR: dispatcher-assisted cardiopulmonary resuscitation; OHCA: out-of-hospital cardiac arrest; ROSC: return of spontaneous circulation; CPC: cerebral performance category; SD: standard deviation. * *p* < 0.05, regarded as significant.

**Table 2:** Performance of dispatcher-assisted cardiopulmonary resuscitation by community socioeconomic status.

OHCA	High SES (*n* = 1656)	Middle SES (*n* = 1025)	Low SES (*n* = 247)	*p* Value
Dispatcher recognition of cardiac arrest (*n*, %)	633 (38.2%)	387 (37.8%)	77 (31.2%)	0.099
Time of dispatcher recognition of cardiac arrest (sec) (mean ± SD)	43.9 ± 35.3	41.7 ± 37.1	64.1 ± 76.4	0.004 *
Early dispatcher recognition of cardiac arrest (≤60 s) (*n*, %)	499 (30.1%)	319 (31.1%)	54 (21.9%)	0.029
Dispatcher initiation of CPR (*n*, %)	563 (34.0%)	332 (32.4%)	69 (27.9%)	0.151
High quality of DA-CPR (*n*, %)	306 (18.5%)	166 (16.2%)	38 (15.4%)	0.215

Abbreviations: SES: socioeconomic status; CPR: cardiopulmonary resuscitation; DA-CPR: dispatcher-assisted cardiopulmonary resuscitation. * *p* < 0.05, regarded as significant.

**Table 3:** Characteristics in patients with out-of-hospital cardiac arrest by achievement of a sustained ROSC or not.

OHCA	Sustained ROSC (*n* = 571)	Non-sustained ROSC (*n* = 2357)	*p* Value
Age (years) (mean ± SD)	66.5 ± 16.0	70.0 ± 16.3	<0.001
Age (> 65 years) (*n*, %)	311 (54.5%)	1493 (63.4%)	<0.001
Male (*n*, %)	353 (61.8%)	1514 (64.2%)	0.282
High SES group (*n*, %)	359 (62.9%)	1297 (55.0%)	0.001
Middle SES group (*n*, %)	174 (30.5%)	851 (36.1%)	0.011
Low SES group (*n*, %)	38 (6.7%)	209 (8.9%)	0.088
Response time (min) (mean ± SD)	6.8 ± 3.1	7.5 ± 3.8	<0.001
Scene time (min) (mean ± SD)	9.7 ± 4.8	10.7 ± 7.4	0.001
Transportation time (min) (mean ± SD)	7.2 ± 5.9	7.9 ± 5.9	0.021
Dispatcher recognition of cardiac arrest (*n*, %)	162 (28.4%)	935 (39.7%)	<0.001
Time of dispatcher recognition of cardiac arrest (sec) (mean ± SD)	50.8 ± 49.9	43.4 ± 38.5	0.032
Dispatcher initiation of CPR (*n*, %)	144 (25.2%)	820 (34.8%)	<0.001
High quality of DA-CPR (*n*, %)	84 (14.7%)	426 (18.1%)	0.057
Witnessed (*n*, %)	390 (68.3%)	1087 (46.1%)	<0.001
Public location (*n*, %)	82 (14.4%)	175 (7.4%)	<0.001
Epinephrine use (*n*, %)	41 (7.2%)	99 (4.2%)	0.003
LMA use (*n*, %)	452 (79.2%)	1917 (81.3%)	0.236
Shockable (*n*, %)	128 (22.4%)	289 (12.3%)	<0.001
Bystander CPR (*n*, %)	176 (30.8%)	641 (27.2%)	0.083
Prehospital ROSC (*n*, %)	80 (14.0%)	39 (1.7%)	<0.001
Ever ROSC (*n*, %)	545 (95.4%)	168 (17.1%)	<0.001
Survival to discharge (*n*, %)	201 (35.2%)	17 (0.7%)	<0.001
Good neurologic outcome at discharge (*n*, %)	74 (13.0%)	2 (0.1%)	<0.001

Abbreviations: SES: socioeconomic status; CPR: cardiopulmonary resuscitation; DA-CPR: dispatcher-assisted cardiopulmonary resuscitation; LMA: laryngeal mask airway; ROSC: return of spontaneous circulation; SES: socioeconomic status.

**Table 4:** Analysis of sustained return of spontaneous circulation.

OHCA	Unadjusted Odds Ratio (95% CIs)	*p* Value	Adjusted Odds Ratio (95% CIs)	*p* Value
Age (>65 y/o)	0.69 (0.58–0.83)	<0.001 *		
Male	0.90 (0.75–1.09)	0.282		
High SES group	1.38 (1.15–1.67)	<0.001 *	1.54 (1.06–2.23)	0.024 *
Middle SES group	0.78 (0.64–0.95)	0.011 *		
Low SES group	0.73 (0.51–1.05)	0.088		
Response time (≤5 min)	1.47 (1.21–1.78)	<0.001 *	1.50 (1.04–2.16)	0.031 *
Scene time (≤8 min)	1.53 (1.26–1.84)	<0.001 *		
Transportation time (≤5 min)	1.25 (1.03–1.50)	0.021 *		
Dispatcher recognition of cardiac arrest	0.60 (0.49–0.74)	<0.001 *		
Early dispatcher recognition of cardiac arrest (≤60 s)	0.63 (0.44–0.92)	0.015 *		
Dispatcher initiation of CPR	0.63 (0.51–0.78)	<0.001 *		
High quality of DA-CPR	0.78 (0.61–1.01)	0.057		
Witnessed	2.52 (2.07–3.06)	<0.001 *	1.63 (1.15–2.32)	0.007 *
Public location	2.09 (1.58–2.77)	<0.001 *		
Epinephrine use	1.76 (1.21–2.57)	0.003 *		
LMA use	0.87 (0.70–1.09)	0.236		
Shockable rhythm	2.07 (1.64–2.61)	<0.001 *	3.49 (2.30–5.30)	<0.001 *
Bystander CPR	1.19 (0.98–1.46)	0.083	1.45 (1.03–2.06)	0.036 *

Abbreviations: CPR: cardiopulmonary resuscitation; CIs: confidence intervals; DA-CPR: dispatcher-assisted cardiopulmonary resuscitation; LMA: laryngeal mask airway; ROSC: return to spontaneous circulation; SES: socioeconomic status. * *p* < 0.05, regarded as significant.

## Data Availability

The data presented in this study was mainly obtained from the Tainan City Fire Bureau and are available with the permission of the Tainan City Fire Bureau.

## Appendix A

**Table A1:** Characteristics of the high, middle, and low socioeconomic status (SES) groups.

OHCA	High SES ^a^ *n* = 10	Middle SES ^b^ *n* = 17	Low SES ^c^ *n* = 10	*p* Value
Average price of real state (US dollars/m^2^)	2740.9 ± 2251.6	285.9 ± 191.2	55.9 ± 22.1	<0.001
Population (10^3^ persons)	123.1 ± 60.9	30.8 ± 12.4	13.2 ± 7.1	<0.001
Area (km^2^)	36.1 ± 30.6	53.9 ± 22.9	91.4 ± 37.6	0.001
Population density (10^3^ persons/km^2^)	6.2 ± 5.1	0.6 ± 0.3	0.2 ± 0.1	<0.001
Mean annual household income (10^3^ US dollars)	30.5 ± 4.0	25.3 ± 2.7	22.5 ± 0.9	<0.001

Note: ^a^. the districts of West Central, Rende, North, Yongkang, Anping, Annan, East, South, Xinying, and Guiren. ^b^. the districts of Xiaying, Shanshang, Baihe, Anding, Xigang, Jiali, Guantian, Houbi, Liuying, Jiangjun, Madou, Shanhua, Xinhua, Xinshi, Xuejia, Guanmiao, and Yanshui. ^c^. the district of Qigu, Danei, Liujia, Beimen, Zuozhen, Yujing, Dongshan, Nanhua, Nanxi, and Longqi.

**Table A2:** Analysis of the obstacles that impeded dispatcher-assisted cardiopulmonary resuscitation for patients with out-of-hospital cardiac arrest.

OHCA	High SES *n* = 1656	Middle SES *n* = 1025	Low SES *n* = 247	*p* Value
Dispatcher can’t recognize need for CPR	320 (19.3%)	194 (18.9%)	48 (19.4%)	0.964
Difficult access to the victim	125 (7.5%)	77 (7.5%)	14 (5.7%)	0.561
CPR is already ongoing	84 (5.1%)	60 (5.9%)	5 (2.0%)	0.049
Overly distraught	54 (3.3%)	33 (3.2%)	8 (3.2%)	0.998
Third party caller	23 (1.4%)	11 (1.1%)	1 (0.4%)	0.473
Patient’s status changed	15 (0.9%)	7 (0.7%)	3 (1.2%)	0.579
Dangerous scene	6 (0.4%)	5 (0.5%)	2 (0.8%)	0.463
Apparent death	9 (0.5%)	2 (0.2%)	0	0.307
Caller unable to move the patient	5 (0.3%)	6 (0.6%)	0	0.445
Language barriers	1 (0.1%)	2 (0.2%)	0	0.664
Valid DNAR orders	1 (0.1%)	1 (0.1%)	0	1

Abbreviations: SES, socioeconomic status; CPR, cardiopulmonary resuscitation; DNAR, do-not-attempt-resuscitation.
